# Compound Heterozygosity for *OTOA* Truncating Variant and Genomic Rearrangement Cause Autosomal Recessive Sensorineural Hearing Loss in an Italian Family

**DOI:** 10.3390/audiolres11030041

**Published:** 2021-09-09

**Authors:** Rocco Pio Ortore, Maria Pia Leone, Orazio Palumbo, Antonio Petracca, Eleonora M. C. Trecca, Aurelio D’Ecclesia, Ciro Lucio Vigliaroli, Lucia Micale, Francesco Longo, Salvatore Melchionda, Marco Castori

**Affiliations:** 1 Division of Maxillofacial Surgery and Otolaryngology, Fondazione IRCCS-Casa Sollievo della Sofferenza, San Giovanni Rotondo, 71013 Foggia, Italy; r.ortore@operapadrepio.it (R.P.O.); a.decclesia@operapadrepio.it (A.D.); l.vigliaroli@operapadrepio.it (C.L.V.); f.longo@operapadrepio.it (F.L.); 2 Division of Medical Genetics, Fondazione IRCCS-Casa Sollievo della Sofferenza, San Giovanni Rotondo, 71013 Foggia, Italy; mp.leone@operapadrepio.it (M.P.L.); o.plaumbo@operapadrepio.it (O.P.); a.petracca@operapadrepio.it (A.P.); l.micale@operapadrepio.it (L.M.); s.melchionda@operapadrepio.it (S.M.); m.castori@operapadrepio.it (M.C.)

**Keywords:** autosomal recessive, deafness, microdeletion, *OTO*, otoancorin

## Abstract

Hearing loss (HL) affects 1–3 newborns per 1000 and, in industrialized countries, recognizes a genetic etiology in more than 80% of the congenital cases. Excluding *GJB2* and *GJB6*, *OTOA* is one of the leading genes associated with autosomal recessive non-syndromic HL. Allelic heterogeneity linked to *OTOA* also includes genomic rearrangements facilitated by non-allelic homologous recombination with the neighboring *OTOAP1* pseudogene. We present a couple of Italian siblings affected by moderate to severe sensorineural hearing loss (SNHL) due to compound heterozygosity at the *OTOA* locus. Multigene panel next-generation sequencing identified the c.2223G>A, p.(Trp741*) variant transmitted from the unaffected mother. Assuming the existence of a second paternal deleterious variant which evaded detection at sequencing, genomic array analysis found a ~150 Kb microdeletion of paternal origin and spanning part of *OTOA*. Both deleterious alleles were identified for the first time. This study demonstrates the utility of an integrated approach to solve complex cases and allow appropriate management to affected individuals and at-risk relatives.

## 1. Introduction

Hearing loss (HL) is a global health issue involving 1 to 3 newborns per 1000 worldwide [1]. Etiology of HL is highly heterogeneous including both acquired and inherited causes. In developed countries, more than 80% of the congenital cases are genetic, therefore having the potential of affecting multiple family members [2]. The impact of HL on the quality of life of affected individuals is variable and mostly influenced by age at onset and severity. Pre-lingual HL may significantly impact the neurodevelopmental trajectory of the affected individual by impairing cognitive functions and social competences. At the same time, HL is a treatable condition by a variety of interventions, including surgical procedures, hearing aids and cochlear implants, which are guided by the underlying pathogenesis and patient’s characteristics. For these reasons, early diagnosis and etiological classification of HL are both crucial for optimal treatment of the index cases as well as relatives at risk.

Among the cases of hereditary HL, about 70% of the instances are isolated presentations (i.e., non-syndromic HL), while the remaining 30% occur within multisystem disorders (i.e., syndromic HL) [3]. Both groups feature extreme genetic heterogeneity. For non-syndromic HL, the Hereditary Hearing Loss Website currently reports 77 genes associated with autosomal recessive non-syndromic HL, 51 genes for autosomal dominant non-syndromic HL and five genes for X-linked non-syndromic HL (https://hereditaryhearingloss.org/, accessed on 2 August 2021). Up to the present time, no less than 600 different syndromes are associated with HL [4]. Therefore, once *GJB2* is excluded (which may account for up the 80% of the cases of autosomal recessive HL) [5], an effective approach to the diagnosis of hereditary HL should consider a wide array of genes, modes of inheritance and molecular mechanisms. In this scenario, the introduction of next-generation sequencing (NGS) technologies, the implementation of dedicated bioinformatics pipelines and the integration with techniques validated for the diagnosis of copy number variants (CNV) have opened a new scenario for the management of families with hereditary HL.

*OTOA* (NM_144672) is located at the 16p12.1 cytogenetic band and is composed of 28 exons. Biallelic variants in *OTOA* cause autosomal recessive non-syndromic deafness 22 (DFNB22) [MIM #607039], which is usually characterized by severe-to-profound deafness involving all frequencies. *OTOA* encodes for otoancorin, which belongs to a group of non-collagenous glycoproteins specifically expressed in the ear of vertebrates. Otoancorin is located at the interface between the apical surface of epithelial cells and the overlying acellular gels of the inner ear [6]. Five different isoforms are deposited in UniProt and the longest isoform (no. 1) includes 1153 amino acids.

We report a pedigree with two affected siblings with autosomal recessive non-syndromic HL due to compound heterozygosity in *OTOA*. This report exemplifies the power of an integrated laboratory approach to resolve molecularly complex cases and expands the molecular repertoire of *OTOA*. 

## 2. Materials and Methods

### 2.1. Family Enrollment and Sample Preparation

The proband and her younger affected brother were enrolled in the routine activities of the Audiology Outpatient Clinic at Fondazione IRCCS-Casa Sollievo della Sofferenza (San Giovanni Rotondo, Italy). The family was subsequently referred to the Medical/Clinical Genetics Service at the same Institution. This family provided written informed consent to molecular testing and to the full content of this publication. This study was conducted in accordance with the 1984 Declaration of Helsinki and its subsequent revisions. The results of this work were entirely obtained by diagnostic procedures; therefore, Institutional Review Board approval was not requested. Peripheral blood samples were collected from the proband, her brother and both parents, and genomic DNA was isolated by using Bio Robot EZ1 (Quiagen, Solna, Sweden). The quality of DNA was tested on 1% electrophorese agarose gel, and the concentration was quantified by Nanodrop 2000 C spectrophotometer (Thermo Fisher Scientific, Waltham, MA, USA).

### 2.2. Next-Generation Sequencing (NGS) Analysis

After DNA extraction and quantification following standard procedures, proband’s DNA was sequenced with a custom-made HaloPlex gene panel (Agilent Technologies, Santa Clara, CA, USA) designed to selectively capture known genes associated to syndromic and nonsyndromic forms of hereditary HL including: *ACTG1* (NM_001199954.1), *BDP1* (NM_018429.2), *CCDC50* (NM_174908.3), *CDH23* (NM_001171930.1), *CEACAM16* (NM_001039213.3), *CLDN14* (NM_001146077.1), *COCH* (NM_001135058.1) *COL11A2* (NM_080680), *CRYM* (NM_001888.4), *DFNA5* (NM_001127453.1), *DFNB31* (NM_001083885.2) *DFNB59* (PJVK) (NM_001042702.3), *DIAPH1* (NM_001079812.2), *ESPN* (NM_031475.2), *ESRRB* (NM_004452.3), *EYA1* (NM_000503.5), *EYA4* (NM_001301012.1), *GIPC3* (NM_133261.2), *GJA1* (NM_000165.4), *GJB2* (NM_004004.5), *GJB3* (NM_001005752.1), *GJB4* (NM_153212.2), *GJB6* NM_001110219.2), *GPSM2* (NM_001321038.1), *GRHL2* (NM_001330593.1), *GRXCR1* (NM_001080476.2) *HGF* (NM_000601.5), *KCNQ4* (NM_004700.3), *LHFPL5* (NM_182548.3), *LOXHD1* (NM_001145472.2), *LRTOMT* (NM_001145307.4), *MARVELD2* (NM_001038603.2), *MITF* (NM_000248.3), *MSRB3* (NM_001031679.2), *MYH14* (NM_001077186.1), *MYH9* (NM_002473.5), *MYO15A* (016239.3), *MYO1A* (NM_001256041.1), *MYO1C* (NM_001080779.1), *MYO1F* (NM_ 012335.3), *MYO3A* (NM_017433.4), *MYO6* (NM_001300899.1), *MYO7A* (NM_000260.3), *OTOA* (NM_001161683.1), *OTOF* (NM_001287489.1), *PAX3* (NM_000438.5), *PCDH15* (NM_001142763.1), *PDZD7* (NM_001195263.1), *POU3F4* (NM_000307.4), *POU4F3* (NM_002700.2), *PRPS1* (NM_001204402.1), *PTPRQ* (NM_001145026.1), *SERPINB6* (NM_001195291.2), *SIX1* (NM_005982.3), *SLC17A8* (NM_001145288.1), *SLC26A4* (NM_000441.1), *SLC26A5* (NM_001167962.1), *SMPX* (NM_014332.2), *SNAI2* (NM_003068.4), *SOX10* (NM_006941.3), *STRC* (NM_153700.2), *TECTA* (NM_005422.2), *TJP2* (NM_001170414.2), *TMC1* (NM_138691.2), *TMIE* (NM_147196.2), *TMPRSS3* (NM_001256317.1), *TMPRSS5* (NM_001288749.1), *TPRN* (NM_001128228.2), *TRIOBP* (NM_001039141.2), *USH1C* (NM_001297764.1), *WFS1* (NM_001145853.1). Libraries were arranged using HaloPlex Target enrichment kit (Agilent Technologies, Santa Clara, CA, USA) following the manufacturer’s instructions. Targeted fragments were then sequenced on MiSeq Desktop Sequencer (Illumina, San Diego, CA, USA) using MiSeq Reagent Kit V3 (Illumina, San Diego, CA, USA).

FastQC files were checked for quality and trimmed, mapped reads recalibrated and processed, and variants annotated by the Alissa Align & Call bioinformatics pipeline (Agilent Technologies, Santa Clara, CA, USA). Annotated variants were then filtered and interpreted with an internally implemented variant triage system by Alissa Interpret (Agilent Technologies, Santa Clara, CA, USA). Variants were first prioritized following these conditions: (i) nonsense/frameshift variant in genes previously described as disease-causing by haploinsufficiency or loss-of-function; (ii) missense variant with a REVEL score ≥0.75; (iii) variant affecting canonical splicing sites (i.e., ±1 or ±2 positions); (iv) variant absent in allele frequency population databases; (v) variant reported in allele frequency population databases, but with a minor allele frequency (MAF) lower than 0.05; (vi) variant predicted and/or annotated as pathogenic/deleterious in ClinVar and/or LOVD without evidence of conflicting interpretation.

### 2.3. Sanger Sequencing

The presence of the candidate variant identified by NGS was confirmed by Sanger sequencing on the proband’s and relatives’ DNA. The primers were designed by using primer3 tool (https://primer3.ut.ee/, accessed on 1 April 2020) to amplify *OTOA* (NM_144672) exon 20 flanking sequences and verified both by BLAST and BLAT against the human genome to ensure specificity (*OTOA*_ex20F: 5′-TCAAAACTCCCAGGGATGAC, *OTOA*_ex20R: 5′ CCTTTTCCAGAACCTTGCAC). The amplified products were subsequently purified by using ExoSAP-IT PCR Product Cleanup Reagent (Thermofisher Scientific, Wilmington DE, USA) and sequenced by using BigDye Terminator v1.1 sequencing kit (Thermofisher Scientific, Wilmington DE, USA). The fragments obtained were purified using DyeEx plates (Qiagen, Tübingen, Germany) and resolved on ABI Prism 3130 Genetic Analyzer (Thermofisher Scientific, Wilmington, DE, USA). Sequences were analyzed using the Sequencer software (Gene Codes, Ann Arbor, MI, USA). The *OTOA* variant has been submitted to the LOVD (Leiden Open Variation Database, https://databases.lovd.nl/shared/individuals/00377583, accessed on 28 July 2021, individual ID #00377583).

### 2.4. Variant Designation and Clinical Interpretation

Nucleotide variant nomenclature follows the format indicated in the Human Genome Variation Society (HGVS, http://www.hgvs.org, accessed on 2 August 2021) recommendations. DNA variant numbering system refers to cDNA. Nucleotide numbering uses +1 as the A of the ATG translation initiation codon in the reference sequence, with the initiation codon as codon 1. For clinical interpretation, SNVs and short insertion/deletion/indels were classified according to the American College of Medical Genetics and Genomics (ACMG)/Association for Molecular Pathology (AMP) recommendations [7] and following integrations by the Sequence Variant Interpretation Working Group (https://clinicalgenome.org/working-groups/sequence-variant-interpretation/, accessed on 2 August 2021) and the expert specification of the ACMG/AMP variant interpretation guidelines for genetic HL [8]. Default criteria have been set with Varsome (https://varsome.com/, accessed on 2 August 2021). In silico prediction criteria (PP3, BP4) were reviewed according to internal bioinformatics pipelines. Criteria associated with family study (i.e., origin in sporadic cases, co-segregation with the phenotype in multiple family members, occurrence in an established unaffected adult individual and lack of segregation in additional affected family members; PS2, PM6, PP1, BS2, BS4) were assigned manually after extended family study. The strength of each criterion was not changed.

### 2.5. Conservation of OTOA p.Trp741 Amino Acid 

Evolutionary conservation of the tryptophan at the 741 position of otoancorin (NP_653273.3), encoded by *OTOA*, was investigated with protein sequence alignment generated by Clustal Omega (https://www.ebi.ac.uk/Tools/msa/clustalo/, accessed on 2 August 2021). The otoancorin amino acidic sequences of indicated species were downloaded from UniProt (https://www.uniprot.org/, accessed on 2 August 2021).

### 2.6. Genomic Array Analysis

In search of a second deleterious variant in OTOA in the proband and affected brother, high-resolution single nucleotide polymorphism-array (SNP-array) analyses of the proband, her brother and parents were executed using the CytoScan HD Array (Thermo Fisher Scientific, Waltham, MA, USA), as previously described [9]. This array contains more than 2.6 million markers for copy number variation (CNVs) analysis and approximately 750,000 SNP probes capable of genotyping with an accuracy greater than 99%. Data analysis was performed using the Chromosome Analysis Suite Software version 4.2 (Thermo Fisher Scientific, Waltham, MA, USA) following a standardized pipeline. Briefly: (i) the raw data file (CEL) was normalized using the default options; (ii) an unpaired analysis was performed using as baseline 270 HapMap samples in order to obtain copy numbers value, while the amplified and/or deleted regions was detected using a standard Hidden Markov Model (HMM) method. We retained CNVs >15 Kb in length and overlapping >10 consecutive probes to reduce the detection of false-positive calls. The significance of each detected CNV was determined by comparing all chromosomal alterations identified in the patient with those collected in an internal database of ~5000 patients studied by SNP arrays since 2010, and public databases including Database of Genomic Variants (DGV), DECIPHER, and ClinVar. Base pair positions, information about genomic regions and genes involved by CNVs, and known associated diseases have been derived from the University of California Santa Cruz (UCSC) Genome Browser, build GRCh37 (hg19). The clinical significance of each rearrangements detected has been assessed following the ACMG guidelines for CNVs reporting [10,11].

## 3. Results

### 3.1. Clinical Report

The proband was a 2-year-old girl referred to our tertiary care Audiology Outpatient Clinic because of failed newborn hearing screening with both transient otoacoustic emissions (TOAEs) and automated auditory brainstem response (A-ABR), and increased threshold at auditory brainstem response (ABR). She was born at term (38 weeks + 5 days) from an uneventful pregnancy and healthy, unrelated parents. The birthweight was 3170 g. No birth defects were identified. The neonatal period was normal. Infectious screening was performed through polymerase chain reaction (PCR) test and a Cytomegalovirus infection was ruled out. Clinical genetics physical exam excluded any facial dysmorphism and any other external structural anomaly. Psychomotor development was otherwise within normal limits. 

She underwent clinical ear, nose and throat (ENT) examination and audiological assessment to determine the type and grade of hearing impairment: conditioned play audiometry (CPA), tympanometry, stapedial reflexes, ABR and TOAEs.

**Figure 1:**
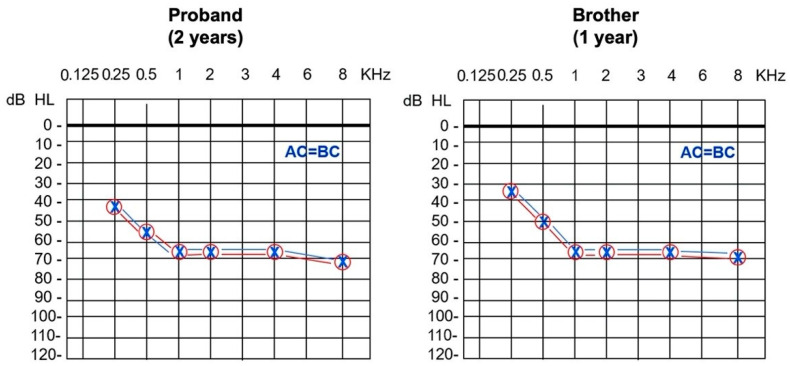
Pure-tone audiometry showing bilateral symmetric sensorineural hearing loss of moderate to severe degree in both the proband, a 2-year-old girl, and her 1-year-old affected brother (AC indicates air conduction; BC, bone conduction).

Clinical ENT examination was normal. Click-evoked ABR showed a threshold for frequencies 2–4 KHz of 65 dB hearing level in both ears. Conditioned play audiometry (CPA) revealed bilateral symmetric SNHL of moderate to severe degree with a pure-tone-average (PTA) for frequencies 0.25, 0.5, 1, 2 and 4 KHz corresponding to a 60 dB hearing level (Figure 1). Tympanometry was normal with absent stapedial reflexes at high frequency stimulations. TOAEs were absent. Computed tomography and magnetic resonance imaging of the inner ear and brain resulted normal. Hearing rehabilitation was obtained with hearing aid fitting associated to speech therapy, with good functional outcomes. Sanger sequencing for *GJB2* point variants and *GJB6* recurrent deletion resulted negative at a different center.

More recently, her 1-year-old brother presented similarly abnormal results at auditory newborn screening program. Click-evoked ABR showed a threshold for frequencies 2–4 KHz of 65 dB hearing level bilaterally, while CPA revealed bilateral symmetric SNHL of moderate to severe degree with a PTA for frequencies 0.25, 0.5, 1, 2 and 4 KHz corresponding to 55 dB hearing level (Figure 1). 

Given the recurrence of the disease in two siblings of different sexes and from unaffected parents, autosomal recessive inheritance was assumed, and second-level molecular testing was performed. 

The last audiological evaluation in February 2021 revealed a stable pure tone threshold and good hearing aid performance in both siblings.

### 3.2. Molecular Findings

**Figure 2:**
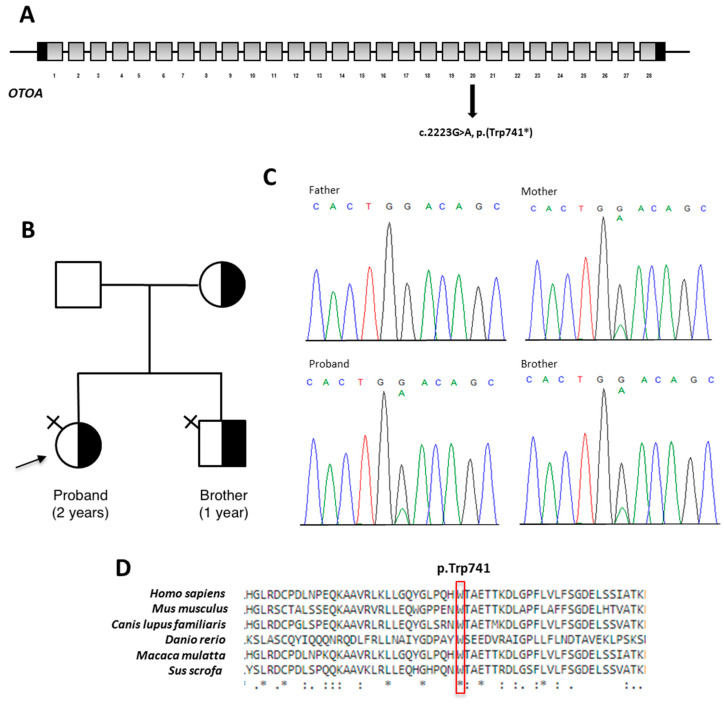
Results of the NGS analysis. (**A**): schematic representation of *OTOA* gene: coding regions are in grey; UTR sequences are in black; introns are not to scale. *OTOA* variant identified here is shown on the gene structure. All of the 5 alternate splice isoforms terminate in exons downstream of exon 20. (**B**): Family pedigree. Black arrow indicates proband. Half-black symbol indicates carrier. Symbol with black cross on the upper right indicates affected individual. (**C**): electropherograms showing DNA sequencing analysis of PCR product amplified with primers targeting *OTOA* exon 20 of the proband’s and her relatives’ DNA. (**D**): Conservation of the region spanning the residue p.Trp741 among species (red box) generated by Clustal Omega.

Targeted NGS analysis performed on patient’s DNA revealed a heterozygous nonsense c.2223G>A variant located in the exon 20 of *OTOA*, which is predicted to incorporate a premature termination codon (PTC) [p.(Trp741*)] (Figure 2A). No further clinically relevant variants were detected in the remaining genes included in the panel. The c.2223G>A, p.(Trp741*) variant is not reported in major databases, including dbSNP, ExAC, 1000 Genomes and gnomAD. This suggests that the variant represents a rare event. The result was confirmed by direct Sanger sequencing of proband’s DNA. Segregation analysis in both unaffected parents revealed that this variant was inherited from the mother. The heterozygous variant was also identified in the affected brother (Figure 2B,C). Computational analysis predicted the pathogenic effect of the novel change which occurs in an evolutionarily conserved region (Figure 2D). According to the ACMG guidelines, the variant was classified as pathogenic by the attribution of the following criteria: PVS1_very strong, PM2_moderate and PP1_supporting [7].

**Figure 3:**
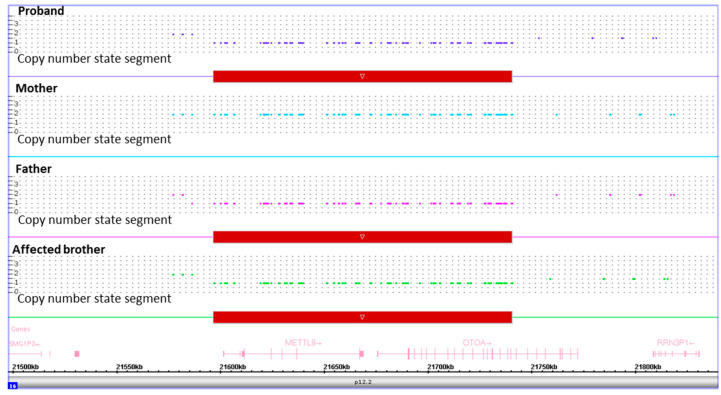
Results of SNP-array analysis in the proband, her affected brother and her unaffected parents. Copy number state of each probe is drawn along chromosome 16 from 21.5 to 21.8 Mb (UCSC Genome Browser, build GRCh37/hg19). The upper panel represents the copy number state of the proband, the middle panels that of the mother and father, and the lower panel that of the affected brother. Values of Y-axis indicate the inferred copy number according to the probe’s intensities. Red bar indicates the deleted region identified in the proband, her father and her affected brother.

SNP-array analysis showed an interstitial microdeletion of ~150 Kb involving the 16p12.2 chromosome region in the proband, affected brother and unaffected father. The deleted region was covered by 168 SNP array probes. This microdeletion encompasses exons 1-19 and part of exon 20 of *OTOA*, as well as the neighboring *METTL9*. Apart from known polymorphisms, no other CNVs were detected. The molecular karyotype of the identified rearrangement is arr[GRCh37] 16p12.2(21585792x2,21596300_21740274x1,21761405x2)pat in accordance with the International System for Human Cytogenetic Nomenclature (ISCN 2020). The same analysis resulted negative in the mother. This further investigation demonstrated that the recurrence of HL in the proband and her younger brother was due to compound heterozygosity for the maternal *OTOA* c.2223G>A, p.(Trp741*) pathogenic variant and the paternal ~150 Kb microdeletion involving part of *OTOA* (Figure 3). Of note, the deleted segment does not overlap the heterozygous variant. For these reason, molecular analysis in the proband and affected brother was not compatible with hemizygosity. 

## 4. Discussion

Here, we reported an Italian family with two siblings affected by non-syndromic SNHL due to compound heterozygosity for *OTOA* deleterious variants. The case was resolved by applying a multi-technique laboratory approach demonstrating a maternal deleterious variant at NGS analysis and, subsequently, revealing a paternal *OTOA* intragenic microdeletion by SNParray.

In Simple ClinVar (https://www.simple-clinvar.broadinstitute.org, accessed on 2 August 2021), 115 distinct sequence variants have been deposited for *OTOA*. Among them, 23 were described as “pathogenic” (#14) or “likely pathogenic” (#9) and only three of them are missense changes, while the remaining are predicted null alleles (i.e., canonical splice site and nonsense variants, intragenic deletions and indels) (last consultation: 31 July 2021). The novel variant c.2223G>A falls in exon 20 and is predicted to introduce a PTC in position 741. The PTC might elicit nonsense-mediated mRNA decay (NMD), with a variable proportion of the mutated allele acting as a null allele. We did not further investigate the transcriptional effect of the identified variant. However, as the aberrant mRNA terminates the translation at a distance more than 50–55 nucleotides upstream of the last splicing-generated exon–exon junction, we conclude that the mutated transcript may not escape the NMD process [12]. We cannot exclude that at least a proportion of transcripts from the maternal allele can be translated into a shortened protein and then, potentially act under physiological conditions.

The paternally inherited allele has a ~150 Kb microdeletion involving the entire *METTL9* gene and the first 20 exons of *OTOA*. This rearrangement partly overlaps the 110 Kb microdeletion found by Laurent and coll. [13]. Microdeletions of *OTOA* are the second most common type of causative CNVs in hereditary HL [14] with a rate of less than 0.1–0.2% in the general population [15]. *OTOA* microdeletions likely originate from nonallelic homologous recombination [14], which are facilitated by the presence of a neighboring pseudogene called *OTOAP1* with high sequence homology with *OTOA*. Therefore, it is expected that the number of causative alleles due to *OTOA* CNVs will increase in the future, thus supporting the clinical utility of bioinformatics pipelines including *CNV* analysis and/or integrative molecular dosage-sensitive investigations in hereditary HL. The presence of the *OTOAP1* pseudogene prompted us to consider conversion as an alternative molecular mechanism leading to point variants in *OTOA* [13]. Concerning the c.2223G>A heterozygous variant in the maternal allele in this family, the mutated allele c.2223A is not present in the deposited sequence of *OTOAP1*. This argues against the hypothesis that this nonsense change arose from gene conversion in our family.

## 5. Conclusions

In conclusion, this work expands the repertoire of *OTOA* causative variants with a novel nonsense change and a private genomic rearrangement spanning the first 20 exons of the gene. The combination of methodologies used shows an effective approach to decipher pathogenic changes/variants in hereditary HL associated genes and offers effective management of affected individuals and their relatives.

## References

[1] Morton C.C., Nance W.E. (2006). Newborn hearing screening—A silent revolution. N. Engl. J. Med..

[2] Shearer A.E., Smith R.J. (2012). Genetics: Advances in genetic testing for deafness. Curr. Opin. Pediatr..

[3] Vona B., Doll J., Hofrichter M.A.H., Haaf T. (2020). Non-syndromic hearing loss: Clinical and diagnostic challenges. Med. Genet..

[4] Parker M., Bitner-Glindzicz M. (2015). Genetic investigations in childhood deafness. Arch. Dis. Child..

[5] Kenneson A., Van Naarden Braun K., Boyle C. (2002). GJB2 (connexin 26) variants and nonsyndromic sensorineural hearing loss: A HuGE review. Genet. Med..

[6] Zwaenepoel I., Mustapha M., Leibovici M., Verpy E., Goodyear R., Liu X.Z., Nouaille S., Nance W.E., Kanaan M., Avraham K.B. (2002). Otoancorin, an inner ear protein restricted to the interface between the apical surface of sensory epithelia and their overlying acellular gels, is defective in autosomal recessive deafness DFNB22. Proc. Natl. Acad. Sci. USA.

[7] Richards S., Aziz N., Bale S., Bick D., Das S., Gastier-Foster J., Grody W.W., Hegde M., Lyon E., Spector E. (2015). ACMG Laboratory Quality Assurance Committee. Standards and guidelines for the interpretation of sequence variants: A joint consensus recommendation of the American College of Medical Genetics and Genomics and the Association for Molecular Pathology. Genet. Med..

[8] Oza A.M., DiStefano M.T., Hemphill S.E., Cushman B.J., Grant A.R., Siegert R.K., Shen J., Chapin A., Boczek N.J., Schimmenti L.A. (2018). ClinGen Hearing Loss Clinical Domain Working Group. Expert specification of the ACMG/AMP variant interpretation guidelines for genetic hearing loss. Hum. Mutat..

[9] Palumbo O., Palumbo P., Di Muro E., Cinque L., Petracca A., Carella M., Castori M. (2020). A Private 16q24.2q24.3 Microduplication in a Boy with Intellectual Disability, Speech Delay and Mild Dysmorphic Features. Genes.

[10] Kearney H.M., Thorland E.C., Brown K.K., Quintero-Rivera F., South S.T. (2011). Working Group of the American College of Medical Genetics Laboratory Quality Assurance Committee. American College of Medical Genetics standards and guidelines for interpretation and reporting of postnatal constitutional copy number variants. Genet. Med..

[11] Riggs E.R., Andersen E.F., Cherry A.M., Kantarci S., Kearney H., Patel A., Raca G., Ritter D.I., South S.T., Thorland E.C. (2020). Technical standards for the interpretation and reporting of constitutional copy-number variants: A joint consensus recommendation of the American College of Medical Genetics and Genomics (ACMG) and the Clinical Genome Resource (ClinGen). Genet. Med..

[12] Isken O., Maquat L.E. (2007). Quality control of eukaryotic mRNA: Safeguarding cells from abnormal mRNA function. Genes Dev..

[13] Laurent S., Gehrig C., Nouspikel T., Amr S.S., Oza A., Murphy E., Vannier A., Béna F.S., Carminho-Rodrigues M.T., Blouin J.L. (2021). Molecular characterization of pathogenic OTOA gene conversions in hearing loss patients. Hum. Mutat..

[14] Zhang F., Gu W., Hurles M.E., Lupski J.R. (2009). Copy number variation in human health, disease, and evolution. Annu. Rev. Genom. Hum. Genet..

[15] MacDonald J.R., Ziman R., Yuen R.K., Feuk L., Scherer S.W. (2014). The Database of Genomic Variants: A curated collection of structural variation in the human genome. Nucleic Acids Res..

